# Prevalence and Distribution of Rotavirus Genotypes Among Children With Acute Gastroenteritis in Areas Other Than Java Island, Indonesia, 2016–2018

**DOI:** 10.3389/fmicb.2021.672837

**Published:** 2021-05-06

**Authors:** Rury Mega Wahyuni, Takako Utsumi, Zayyin Dinana, Laura Navika Yamani, Juniastuti, Ishak Samuel Wuwuti, Elsa Fitriana, Emily Gunawan, Yujiao Liang, Fitratul Ramadhan, Soetjipto, Maria Inge Lusida, Ikuo Shoji

**Affiliations:** ^1^Indonesia-Japan Collaborative Research Center for Emerging and Re-emerging Infectious Diseases, Institute of Tropical Disease, Airlangga University, Surabaya, Indonesia; ^2^Center for Infectious Diseases, Kobe University Graduate School of Medicine, Kobe, Japan; ^3^Department of Epidemiology, Faculty of Public Health, Campus C, Airlangga University, Surabaya, Indonesia; ^4^Microbiology Division, Sorong Hospital, Sorong, Indonesia; ^5^Institute of Tropical Disease, Airlangga University, Surabaya, Indonesia; ^6^Department of Medical Laboratory, Dompu Hospital, Dompu, Indonesia

**Keywords:** rotavirus, equine-like G3 strain, genotype replacement, children, Indonesia

## Abstract

Group A rotaviruses (RVAs) are the leading cause of acute gastroenteritis, which is often associated with severe symptoms in children under 5 years old. Genetic reassortments and interspecies transmission commonly occur, resulting in a great diversity of RVA circulating in the world. The aim of this study is to determine the prevalence and distribution of RVA genotypes among children in Indonesia over the years 2016–2018 across representative areas of the country. Stool samples were collected from 202 pediatric patients with acute gastroenteritis in three regions of Indonesia (West Nusa Tenggara, South Sumatra, and West Papua) in 2016–2018. Rotavirus G and P genotypes were determined by reverse transcription PCR (RT-PCR) and direct sequencing analysis. The prevalences of RVA in South Sumatra (55.4%) and West Papua (54.0%) were significantly higher than that in East Java (31.7%) as determined in our previous study. The prevalence in West Nusa Tenggara (42.6%) was the lowest among three regions, but higher than that in East Java. Interestingly, equine-like G3 rotavirus strains were found as predominant strains in South Sumatra in 2016 and in West Papua in 2017–2018. Moreover, the equine-like G3 strains in South Sumatra detected in 2016 were completely replaced by human G1 and G2 in 2018. In conclusion, RVA infection in South Sumatra and West Papua was highly endemic. Equine-like G3 strains were also spread to South Sumatra (West Indonesia) and West Papua (East Indonesia), as well as Java Island. Dynamic change in rotavirus genotypes from equine-like G3 to human genotypes was also observed. Continuous monitoring may be warranted in isolated areas in Indonesia.

## Introduction

Diarrheal disease is one of the major causes of morbidity and mortality in children throughout the world, especially in developing countries (Jain et al., 2016; Hakim et al., 2018; Ugboko et al., 2020). Rotavirus A (RVA) is the most common etiologic viral agent causing gastroenteritis in infants and young children worldwide (Chen et al., 2006; Tran et al., 2010; Hakim et al., 2018; Badur et al., 2019; Lestari et al., 2020). In 2016, RVA was responsible for 128,500 deaths globally among children under 5 years old (Troeger et al., 2018). The prevalence of RVA in children in Indonesia was 37.5% over the years 1977–1999, and was increased to 45.5% in 2004–2005 (Putnam et al., 2007; Radji et al., 2010). RVA infection causes severe acute gastroenteritis among children and is responsible for approximately 60% of inpatients and 41% of outpatients in pediatric hospitals, respectively (Nirwati et al., 2017; Utsumi et al., 2018). RVA is responsible for 2% mortality of all causes in children under 5 years old in Indonesia (Parwata et al., 2016).

RVA is a double-stranded RNA virus belonging to the *Reoviridae* family. The RVA genome consists of 11 RNA segments and encodes six structural proteins (VPs) and six non-structural protein (NSPs; Fujii et al., 2019). Based on the antigenicity of the middle layer VP6, rotavirus is serotyped into at least seven species or groups. There are two outer capsid proteins denoted as VP7 (glycoprotein) and VP4 (protease-sensitive) that are used for classification of G and P genotypes, respectively. To date, 36 G-genotypes and 51 P-genotypes have been recognized by the Rotavirus Classification Working Group (2019).^1^ The most common G-types in humans are G1–G4 and G9, and the most common P-types are P[4], P[6], and P[8] (Santos and Hoshino, 2005). The most common G-P combinations are G1P[8], G2P[4], G3P[8], G4P[8], and G9P[8] (Dóró et al., 2014). These genotypes have also been the predominant genotypes circulating in Indonesia (Hakim et al., 2018; Athiyyah et al., 2019).

Previous studies reported that genetic reassortments commonly occur in RVA, resulting in a great diversity of wild-type and interspecies transmission circulating in the world (Matthijnssens et al., 2011; Cowley et al., 2016; Dóró et al., 2016; Komoto et al., 2016; Doan et al., 2017; Utsumi et al., 2018). Indeed, we demonstrated an uncommon RVA genotype, equine-like G3 as a predominant genotype in East Java, Indonesia in 2015–2017. Whole genome sequencing revealed that the predominant strains were inter-genogroup reassortants consisting of equine-like G3 VP7, P[8] VP4, and genogroup 2 backbone I2-R2-C2-M2-A2-N2-T2-E2-H2 (the DS-1-like backbone; Utsumi et al., 2018; Athiyyah et al., 2019). Phylogenetic analyses in VP7 and VP4 revealed that the strains were classified into equine-like G3P[8] and P[6], and the VP7 genes were genetically close to those of the equine strain, which was also found in Yogyakarta, Indonesia in 2018 (Nirwati et al., 2019). However, there have been no reports in other regions of Indonesia. Equine-like G3 strains have also been found in some other countries (Arana et al., 2016; Cowley et al., 2016; Dóró et al., 2016; Guerra et al., 2016; Komoto et al., 2016). In addition to detection of the equine-like G3 genotype, we found a dynamic genotype change from equine-like G3 mentioned above reverting back to typical human G1 with genogroup 1 (Wa-like) constellation (human G1 VP7, P[8] VP4, and genogroup 1 backbone I1-R1-C1-M1-A1-N1-T1-E1-H1) in East Java, Indonesia (Athiyyah et al., 2019). A report from Australia mentioned that equine-like G3P[8] was more common in the regions where the Rotarix® vaccine was used (Roczo-Farkas et al., 2016). Since RVA vaccines have not yet been included in a national immunization program, no data on the impact of RVA vaccination are available in Indonesia. Moreover, studies on the rotavirus genotypes are crucial to obtain fundamental data regarding the genotypes circulating in the pre-vaccination era in Indonesia. This study was conducted to determine the prevalence of RVA in regions other than Java Island, Indonesia and to analyze the RVA genotypes.

## Materials and Methods

### Sample Collection

A total of 202 stool samples were collected from pediatric patients who were hospitalized due to acute gastroenteritis at two hospitals and two health centers in Lampung (South Sumatra), a hospital in Sorong (West Papua), and a hospital in Dompu (West Nusa Tenggara). Seventy-four samples were collected in South Sumatra in 2016 and 2018, 74 were collected in West Papua in 2017–2018, and 54 were in West Nusa Tenggara in 2018. The results from our previous study utilizing the samples collected in East Java in 2015–2018 (Athiyyah et al., 2019) were referenced for comparison in this study. The year and the duration of sample collection depended on the location. We could not collect stool samples in 2017 in South Sumatra because the permission procedure for sampling had been delayed there. The study population ranged in age from birth to 15 years. Subjects were classified into seven age groups: <6, 6–11, 11–23, 24–35, 36–47, 48–59, and ≥60 months. Acute gastroenteritis was defined as the occurrence of ≥3 watery or looser-than normal stools per day for fewer than 14 days. Informed consent for participation was obtained by each parent or guardian of the participants. Ethical approval was obtained from the ethics committees of the participating institutions, i.e., Airlangga University in Indonesia and Kobe University in Japan. The stool samples were collected and stored at −20°C in each facility before being transported to the Institute of Tropical Disease, Airlangga University, Surabaya, Indonesia. Stool samples were transported in refrigerated boxes to the Institute of Tropical Disease, and stored at −80°C until laboratory testing.

### Rotavirus Detection by Immunoassay

All stool samples were tested for the presence of RVA antigen by the immunochromatography method using a Dipstick “Eiken” Rota kit (Eiken Chemical, Tokyo) according to the manufacturer’s instructions. The sensitivity and specificity of Dipstick “Eiken” Rota kit were 95.8 and 93.3%, respectively, when compared with reference reverse transcription PCR (RT-PCR) method (Khamrin et al., 2011).

### RNA Extraction and RT-PCR Genotyping

Ten percent (wt/vol) stool suspensions were prepared with sterile distilled water and clarified by centrifugation at 21,000 × *g* for 10 min. Viral RNA was extracted from 140 μl of the supernatant with a QIAamp Viral RNA mini Kit (Qiagen, Hilden, Germany). RNA was eluted with 60 μl of RNase-, DNase-free water. All samples that were determined to be positive by immunochromatography test were subjected to genotyping in the VP7 (G typing) and VP4 genes (P typing) by multiplex RT-PCR. The VP7 and VP4 primer sets we used had been previously described (Athiyyah et al., 2019). In particular, the VP7 primer set allowed us to correctly identify equine-like G3 among the other epidemic strains (G1, G2, typical human G3, G4, G8, G9, and G12; Fujii et al., 2019).

The RNA samples were initially incubated at 65°C for 5 min with the first PCR primers. Subsequently, reverse transcription reaction was performed at 45°C for 10 min and at 94°C for 2 min, followed by 40 cycles of amplification (at 98°C for 10 s, at 50°C for 15 s, and at 68°C for 40 s), with a final extension at 68°C for 3 min. One μl of diluted (50-fold) first PCR products was then used for a second PCR. The initial denaturation step was conducted at 98°C for 10 min, followed by 20 cycles of amplification (at 98°C for 10 s, at 50°C for 15 s, and at 68°C for 60 s), with a final extension at 68°C for 3 min. Both negative and positive controls were included in each experiment.

### Direct Sequencing and Phylogenetic Analysis

To further investigate RVA genotypes in G and P typing and sequence RVA genomes, all VP7 and VP4 PCR-positive samples were sequenced. The sequences were determined directly from the PCR products with a BigDye terminator ver. 3.1 cycle sequencing kit using an Applied Biosystems 3500XL Genetic Analyzer (Applied Biosystems, Waltham, MA, United States). Phylogenetic analyses were performed based on the nucleotide sequences of the amplified VP7 and VP4 genes by RT-PCR. Representative strains for each G- and P-genotype successfully sequenced were selected for phylogenetic analyses. Reference sequences were retrieved from the DNA Data Bank of Japan/European Molecular Biology Laboratory/GenBank databases. Alignments were performed using the CLUSTAL X (version 1.83) software, and phylogenetic trees were constructed by the neighbor-joining method. To confirm the reliability of phylogenetic tree analysis, bootstrap resampling, and reconstruction were carried out 1,000 times. These analyses were carried out using Molecular Evolutionary Genetic Analysis (MEGA4) software (Tamura et al., 2007). The gene sequences described in the present study have been deposited in the GenBank database under accession numbers LC597263–LC597321 and MW840090–MW840135.

### Statistical Analysis

Statistical analysis was performed using SPSS Statistics 17.0 (Advanced Analytics, Tokyo). Chi-square test or Fisher’s exact test was used to compare between the rotavirus prevalences and locations where samples were collected, sex and age groups. Results were considered statistically significant at *p* < 0.05.

## Results

### Prevalence of RVA Infection

RVA was detected from 104 samples as confirmed by PCR among 105 immunochromatography positive samples, accounting for 55.4% (41 of 74) of pediatric patients in South Sumatra in 2016 and 2018, 50.4% (40 of 74) of pediatric patients in West Papua in 2017–2018, and 42.6% (23 of 54) of pediatric patients in West Nusa Tenggara in 2018, respectively (Table 1).

**Table 1:** Prevalence of rotavirus A (RVA) infection among children with acute gastroenteritis during 2015–2018 in four islands in Indonesia.

Location and year sample collected	Total patients tested	Percentage of patients with rotavirus positive by PCR
Lampung-South Sumatra (2016 and 2018, this study)	74	41 (55.4%)^*^
Sorong-West Papua (2017–2018, this study)	74	40 (54.0%)^**^
Dompu-West Nusa Tenggara (2018, this study)	54	23 (42.6%)
Surabaya-East Java^†^ (2015–2018, previous study)	432	137 (31.7%)^*^^,^^**^

† Athiyyah et al., 2019.

* *p* < 0.01.

** *p* < 0.01.

RVA infections were detected among all age groups in this study. All 202 pediatric patients were analyzed to determine the age- and sex-specific prevalence. As demonstrated in Table 2, RVA infection was most prevalent in the 12–23 month age group, and the RVA prevalence in children <24 months old was significantly higher than those ≥24 months old (*p* < 0.05). A significant difference in RVA prevalence was observed between males and females (*p* < 0.05; Table 2).

**Table 2:** Age- and sex-specific prevalence of rotavirus infection in 2016–2018 in West Nusa Tenggara, South Sumatera, and West Papua.

Characteristics	Number of patients (%) (*n* = 202)^*^	Rotavirus positive (%) (*n* = 104)^**^	
Sex
Male	117 (57.9%)	68 (65.4%)	*p* < 0.05
Female	82 (40.6%)	34 (32.7%)	
NA	3 (1.5%)	2 (1.9%)	
Age (months)
<6	46 (22.8%)	20 (19.2%)	
6–11	47 (23.3%)	26(25.0%)	
12–23	57 (28.2%)	38 (36.5%)	
24–35	18 (8.9%)	6 (5.8%)	*p* < 0.05
36–47	8 (4.0%)	4 (3.9%)
48–59	5 (2.5%)	2 (1.9%)	
≥60	9 (4.4%)	2 (1.9%)	
NA	12 (5.9%)	6 (5.8%)	

* patients with acute gastroenteritis.

** RVA PCR positive.

NA, no data available.

### Distribution and Transition of the RVA G and P Genotypes

**Figure 1:**
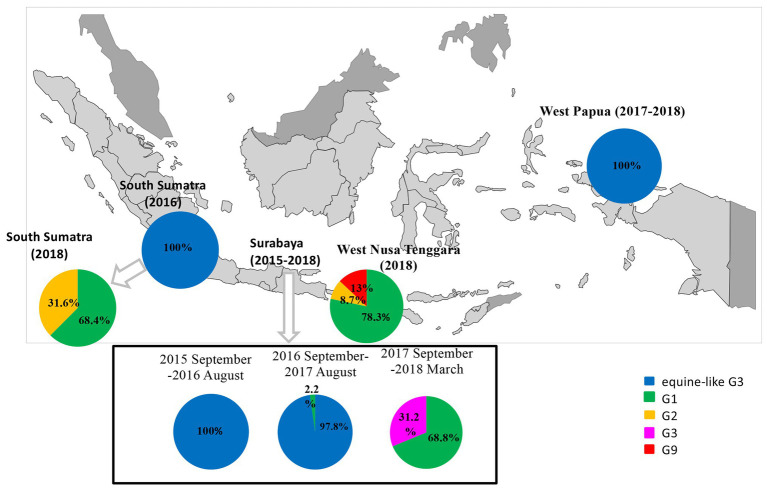
Distribution and change of G-genotypes among children with RVA infections in three islands in Indonesia (West Nusa Tenggara, South Sumatra, and West Papua) in 2016–2018 and East Java as a reference in 2015–2018.

G genotypes were found in 98 of 104 pediatric patients with RVA-related gastroenteritis, while P genotypes were found in 88 cases by PCR. Consequently, G- and P-typing by PCR failed in 16 and 26 cases, respectively. The four RVA G genotypes observed during the study period were human G1, G2, G9, and equine-like G3, and the 3 P genotypes were P[4], P[6], and P[8]. With respect to the G genotypes, in South Sumatra, the equine-like G3 strain was determined to be the dominant genotype (22/22, 100%) in 2016, and this strain was completely replaced by typical human G1 (11/16, 68.8%) and G2 (5/16, 31.2%) in 2018 (data unavailable in 2017; Figure 1). In West Papua, the equine-like G3 genotype was found to be predominant in 2017–2018 (Figure 1). In West Nusa Tenggara, the most prevalent genotype was human G1 (18/23, 78.3%), followed by G9 (3/23, 13.0%,), and G2 (2/23, 8.7%) in 2018 (Figure 1). For the P genotypes, in South Sumatra, P[8] was determined to be the dominant genotype (22/22, 100%) in 2016, while P[4] (7/16, 43.8%) and P[8] were detected (9/16, 56.2%) in 2018. In West Papua, the most prevalent genotype was P[8] (21/23, 91.3%), followed by P[6] (2/23, 8.7%) in 2017–2018. In West Nusa Tenggara, the most prevalent genotype was P[8] (21/23, 91.3%), followed by P[4] (8.7%) in 2018.

A total of 84 samples had both G- and P-type combinations while the remaining 20 only had either one of them successfully typed by PCR. Among them, the most common G-P combinations in West Papua in 2017–2018 were equine-like G3P[8] (21/23, 91.3%), followed by equine-like G3P[6] (2/23, 8.7%); in West Nusa Tenggara in 2018 they were G1P[8] (18/23, 78.3%), followed by G9P[8] (3/23, 13.0%) and G2P[4] (2/23, 8.7%); in South Sumatra in 2016, the most common combination was equine-like G3P[8] (22/22, 100%); and in South Sumatra in 2018, the most common combinations was G1P[8] (9/56, 56.3%), followed by G2P[4] (5/16, 31.2%) and G1P[4] (2/16, 12.5%; Table 3).

**Table 3:** Geographic distribution of rotavirus G and P genotype combinations in Indonesia.

Genotypes	West Papua in 2017–2018	West Nusa Tenggara in 2018	South Sumatra	
			2016	2018
Equine-like G3P[8]	21 (91.3%)	0	22 (100%)	0
Equine-like G3P[6]	2 (8.7%)	0	0	0
G1P[8]	0	18 (78.3%)	0	9 (56.3%)
G1P[4]	0	0	0	2 (12.5%)
G2P[4]	0	2 (8.7%)	0	5 (31.2%)
G9P[8]	0	3 (13.0%)	0	0
Total	23	23	22	16

### Nucleotide Sequencing and Phylogenetic Analysis of RVAs in VP7 and VP4 Genes

**Figure 2:**
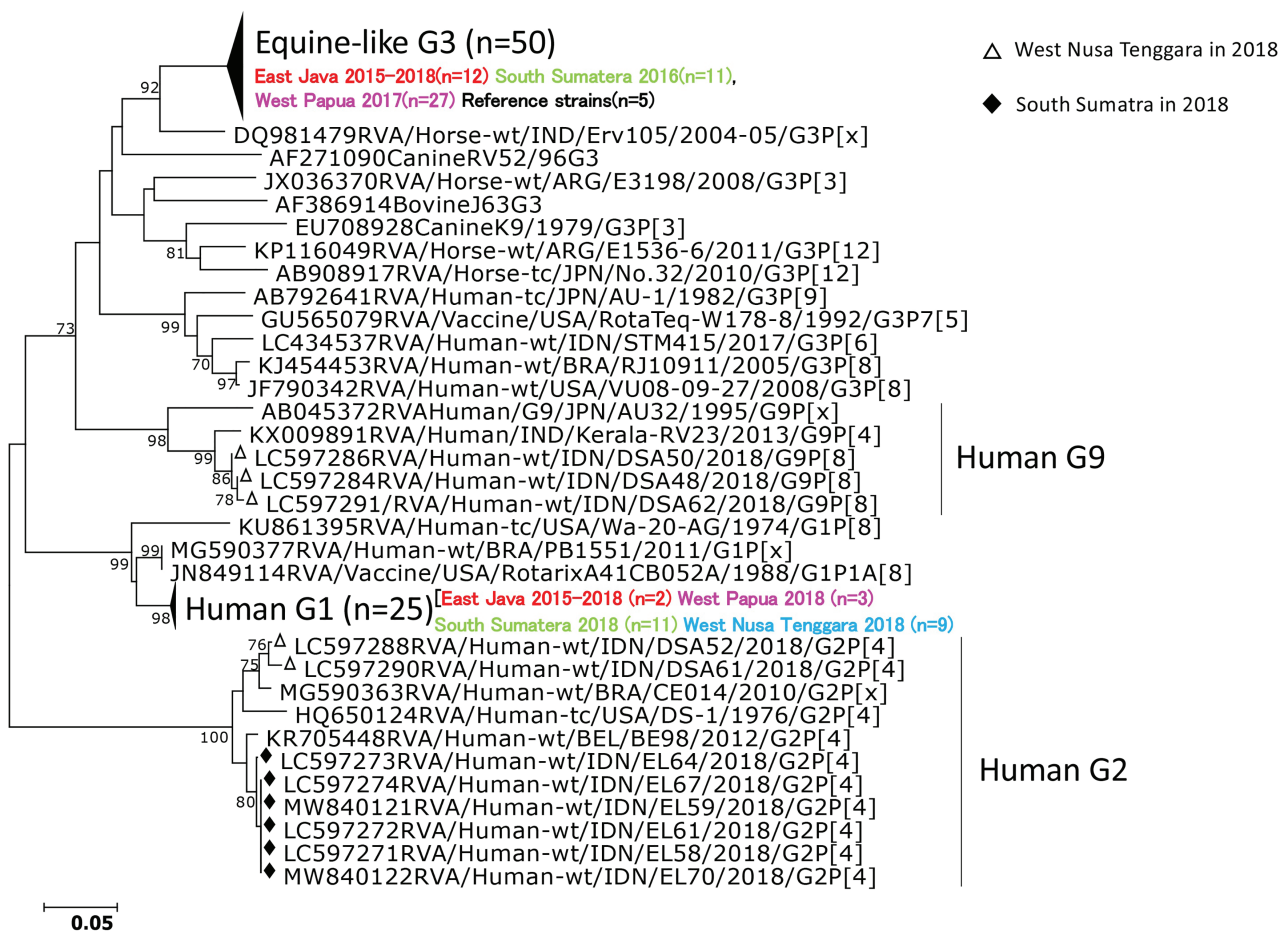
Phylogenetic analysis of the RVA VP7 (G genotype) gene. The tree was constructed using the neighbor-joining method. Bootstrap values (>70) are shown at the branch nodes. The strains in this study are indicated by black diamonds (South Sumatra in 2018), white triangles (West Nusa Tenggara in 2018).

**Figure 3:**
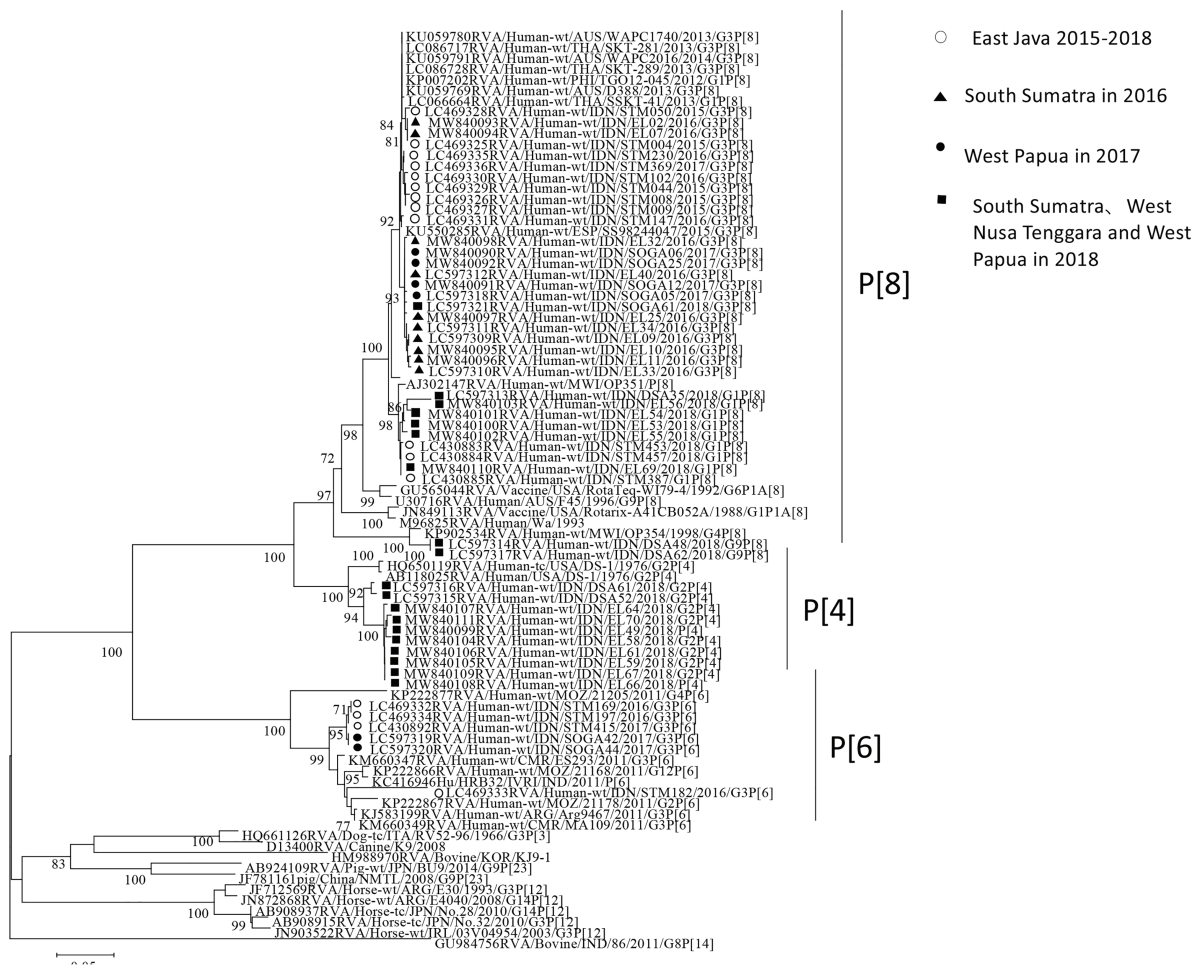
Phylogenetic analysis of the RVA VP4 (P genotype) gene. The tree was constructed using the neighbor-joining method. Bootstrap values (>70) are shown at the branch nodes. The strains in this study are indicated by closed circles (West Papua in 2017), closed triangles (South Sumatra in 2016), closed squares (South Sumatra, West Nusa Tenggara, and West Papua in 2018) and open circles (East Java in 2015–2018).

The partial length of all the strains detected by PCR in VP7 and VP4 were sequenced. Successfully sequenced representative strains were included in the phylogenetic analyses. In the VP7 gene, all of the strains from South Sumatra in 2016 and West Papua in 2017–2018 were classified into equine-like G3 together with the strains from our 2015–2017 study in East Java, Indonesia. Most strains in South Sumatra in 2016 and West Papua in 2017 tended to form their own clusters in the equine-like G3 lineage with 100% nucleotide identities, respectively (Figure 2). The nucleotide sequence identities of the VP7 gene among equine-like G3 strains in South Sumatra in 2016, West Papua in 2017 and East Java and other countries were high (98.2–99.1%). The strains from South Sumatra and West Nusa Tenggara isolated in 2018 belong to human G1, G2, and G9. In the VP4 gene, representative strains in this study were classified into P[8], P[6], and P[4] (Figure 3). The P[6] and P[8] strains in this study were grouped with the equine-like G3P[6] and equine-like G3P[8] strains from East Java in 2015–2017, respectively (Figure 3).

## Discussion

RVA has been the common etiological agent in cases of acute gastroenteritis among children in Indonesia. The unique RVA genotype, equine-like G3P[8]/P[6] with DS-1-like backbone, detected in our previous study by whole genome sequencing became predominant in 2015–2017 (Utsumi et al., 2018; Athiyyah et al., 2019). These findings raise the question of whether the equine-like G3 RVA genotype has spread to other parts of Indonesia. In this molecular epidemiological study, we investigated the RVA infections in regions other than Java Island – namely, South Sumatra (West Indonesia), West Papua (East Indonesia), and West Nusa Tenggara (South East Indonesia).

Interestingly, equine-like G3 rotavirus strains were found to be the predominant strains in South Sumatra in 2016 and in West Papua in 2017–2018. In other words, no other genotype was found in these places in the same years. In addition, the equine-like G3 strains in South Sumatra detected in 2016 were completely replaced by human G1 and G2 in 2018. The combination genotypes distributed in South Sumatra in 2018 were G1P[8] (56.3%), G2P[4] (31.2%), and G1P[4] (12.5%). G1P [4] is an uncommon genotype in Indonesia (Sudarmo et al., 2015). Although the distribution of RVA genotypes in 2017 in South Sumatra remains unclear due to a lack of samples, we have previously demonstrated the evolution of RVA resulted in rapid genotype replacement in East Java (Athiyyah et al., 2019). Dynamic genotype replacement from equine-like G3 to human typical G1 occurred in the middle of 2017 after the dominant circulation of equine-like G3 in 2015–2016 in East Java. We speculated that this phenomenon may have been due to the introduction of the rotavirus vaccine Rotarix (Mukhopadhya et al., 2017; Luchs et al., 2019), because rapid genotype replacement occurred even in regions with low vaccination coverage. However, the introduction of rotavirus vaccines in Indonesia are currently still limited to the private market and data on vaccine coverage in Indonesia are unavailable. Therefore, it is difficult to determine how genotype replacement could simultaneously take place rapidly in West Papua as in East Java. Phylogenetic analyses revealed that equine-like G3 strains from South Sumatra in 2016 and West Papua in 2017 were genetically close to not only strains from East Java, Indonesia but also those from Europe, Australia, and Thailand. These findings highlight the importance of continuous monitoring.

In West Nusa Tenggara, there were no available data for the equine-like RVA genotype in 2018. The globally common combination genotypes of G1P[8], G2P[4], and G9P[8] were detected in West Nusa Tenggara in 2018. G2P[4] was found in the post-vaccination era (Dóró et al., 2014), and this report indicates that this was a regional phenomenon including countries not using the vaccine. An outbreak investigation previously conducted in the adjacent province, East Nusa Tenggara, in 2018 also revealed RVA G2P[4] as the possible causative agent of the acute gastroenteritis outbreak (Utsumi et al., 2020). G1P[8] strains have been reported in many regions of the world (Komoto et al., 2018), including in Indonesia. G9P[8] strains were found in Thailand in 2015–2018 (Fukuda et al., 2020). However, our data are too limited to completely rule out the circulation of equine-like G3P[8] strains in West Nusa Tenggara.

G1P[4] reassortment may have emerged through the introduction of G2P[4] strains (Abdel-Haq et al., 2003). A whole genome study in Vietnam revealed that G1P[4] RVA strains might be generated by genetic reassortment between G1P[8] and G2P[4] RVA strains (Agbemabiese et al., 2017). Since both G1P[8] and G2P[4] co-circulated in the South Sumatra in 2018, G1P[4] in Sumatra may have had genetic reassortment. This speculation, however, requires additional information from whole genome sequencing. On the other hand, both children were from the same village in Sumatra and their samples were collected within the same month, suggesting that there is an epidemiological link that could be traced back in the two G1P[4] strains detected.

Our study showed that the prevalence of RVA infection was significantly higher in South Sumatra and West Papua than in East Java (*p* < 0.01), and the prevalence tended to be higher in West Nusa Tenggara as well. However, the prevalence in South Sumatra, West Papua, and West Nusa Tenggara is similar to those in other parts of Indonesia (Soenarto et al., 2009; Radji et al., 2010; Nirwati et al., 2019). Rotavirus vaccine was introduced in Indonesia in 2006 (Gunawan et al., 2019), but has not been included in the national immunization program of Indonesia. Therefore, the prevalence of RVA infections is still a major health problem among children in Indonesia. In our study, the prevalence of RVA in males was more prevalent than that in females (57.4%; *p* < 0.05). This is consistent with the finding that males are twice more susceptible to rotavirus infection than females and are more likely to be in severe condition (Salim et al., 2014). Approximately 80% of children in this study were <24 months old. There was a significant difference in the prevalence of RVA infection between children <24 months old and children aged ≥24 months (*p* < 0.05) in this study, suggesting that rotavirus vaccine should be administered at an early age after birth. Since the Indonesian government is expected to add the rotavirus vaccine to the national immunization program, it will be very important to evaluate the impact of rotavirus vaccine in the post-vaccination era.

In conclusion, the equine-like G3 strain spread to South Sumatra (West Indonesia) in 2016 and to West Papua (East Indonesia) in 2017–2018 as well as Java Island. Dynamic change in rotavirus genotypes from equine-like G3 to typical human genotypes was also observed. RVA infection in South Sumatra and West Papua is still highly endemic. Our results suggest that continuous monitoring may be warranted in isolated areas in Indonesia.

## Data Availability Statement

The datasets presented in this study can be found in online repositories. The names of the repository/repositories and accession number(s) can be found in the article/supplementary material.

## Ethics Statement

The studies involving human participants were reviewed and approved by Airlangga University, Indonesia Kobe University Graduate School of Medicine, Japan. Written informed consent to participate in this study was provided by the participants’ legal guardian/next of kin.

## Author Contributions

EF collected samples and patient’s data in South Sumatra. IW collected samples and patient’s data in West Papua. FR collected samples and patient’s data in West Nusa Tenggara. TU was responsible for writing the manuscript. RW, ZD, and LY performed all experiments. Soetjipto, Juniastuti, ML, EG, and YL gave assistance for the research. RW and IS gave assistance for the research and analysis. IS supervised the study and helped to draft the manuscript. All authors contributed to the article and approved the submitted version.

### Conflict of Interest

The authors declare that the research was conducted in the absence of any commercial or financial relationships that could be construed as a potential conflict of interest.

## Footnotes

^1^https://rega.kuleuven.be/cev/viralmetagenomics/virus-classification/newgenotypes

## References

[ref1] Abdel-Haq N. M. Thomas R. A. Asmar B. I. Zacharova V. Lyman W. D. (2003). Increased prevalence of G1P[4] genotype among children with rotavirus-associated gastroenteritis in metropolitan Detroit. J. Clin. Microbiol. 41, 2680–2682. 10.1128/JCM.41.6.2680-2682.2003, PMID: 12791903 PMC156486

[ref2] Agbemabiese C. A. Nakagomi T. Nguyen M. Q. Gauchan P. Nakagomi O. (2017). Reassortant DS-1-like G1P[4] rotavirus a strains generated from co-circulating strains in Vietnam, 2012/2013. Microbiol. Immunol. 61, 328–336. 10.1111/1348-0421.12501, PMID: 28696017

[ref3] Arana A. Montes M. Jere K. C. Alkorta M. Iturriza-Gómara M. Cilla G. (2016). Emergence and spread of G3P[8] rotaviruses possessing an equine-like VP7 and a DS-1-like genetic backbone in the Basque Country (North of Spain), 2015. Infect. Genet. Evol. 44, 137–144. 10.1016/j.meegid.2016.06.048, PMID: 27370571

[ref4] Athiyyah A. F. Utsumi T. Wahyuni R. M. Dinana Z. Yamani L. N. Soetjipto S.. (2019). Molecular epidemiology and clinical features of rotavirus infection among pediatric patients in East Java, Indonesia during 2015-2018: dynamic changes in rotavirus genotypes from equine-like G3 to typical human G1/G3. Front. Microbiol. 10:940. 10.3389/fmicb.2019.00940, PMID: 31130934 PMC6510320

[ref5] Badur S. Öztürk S. Pereira P. AbdelGhany M. Khalaf M. Lagoubi Y.. (2019). Systematic review of the rotavirus infection burden in the WHO-EMRO region. Hum. Vaccin. Immunother. 15, 2754–2768. 10.1080/21645515.2019.1603984, PMID: 30964372 PMC6930073

[ref6] Chen S. M. Ni Y. H. Chen H. L. Chang M. H. (2006). Microbial etiology of acute gastroenteritis in hospitalized children in Taiwan. J. Formos. Med. Assoc. 105, 964–970. 10.1016/S0929-6646(09)60280-1 17185238

[ref7] Cowley D. Donato C. M. Roczo-Farkas S. Kirkwood C. D. (2016). Emergence of a novel equine-like G3P[8] inter-genogroup reassortant rotavirus strain associated with gastroenteritis in Australian children. J. Gen. Virol. 97, 403–410. 10.1099/jgv.0.000352, PMID: 26588920

[ref8] Doan Y. H. Suzuki Y. Fujii Y. Haga K. Fujimoto A. Takai-Todaka R.. (2017). Complex reassortment events of unusual G9P[4] rotavirus strains in India between 2011 and 2013. Infect. Genet. Evol. 54, 417–428. 10.1016/j.meegid.2017.07.025, PMID: 28750901

[ref9] Dóró R. László B. Martella V. Leshem E. Gentsch J. Parashar U.. (2014). Review of global rotavirus strain prevalence data from six years post vaccine licensure surveillance: is there evidence of strain selection from vaccine pressure? Infect. Genet. Evol. 28, 446–461. 10.1016/j.meegid.2014.08.017, PMID: 25224179 PMC7976110

[ref10] Dóró R. Marton S. Bartókné A. H. Lengyel G. Agócs Z. Jakab F.. (2016). Equine-like G3 rotavirus in Hungary, 2015- is it a novel intergenogroup reassortant pandemic strain? Acta Microbiol. Immunol. Hung. 63, 243–255. 10.1556/030.63.2016.2.8, PMID: 27352976

[ref11] Fujii Y. Doan Y. H. Wahyuni R. M. Lusida M. I. Utsumi T. Shoji I.. (2019). Improvement of rotavirus genotyping method by using the semi-nested multiplex-PCR with new primer set. Front. Microbiol. 10:647. 10.3389/fmicb.2019.00647, PMID: 30984154 PMC6449864

[ref12] Fukuda S. Tacharoenmuang R. Guntapong R. Upachai S. Singchai P. Ide T.. (2020). Full genome characterization of novel DS-1-like G9P[8] rotavirus strains that have emerged in Thailand. PLoS One 15:e0231099. 10.1371/journal.pone.0231099, PMID: 32320419 PMC7176146

[ref13] Guerra S. Soares L. S. Lobo P. S. Penha Júnior E. T. Sousa Júnior E. C. Bezerra D.. (2016). Detection of a novel equine-like G3 rotavirus associated with acute gastroenteritis in Brazil. J. Gen. Virol. 97, 3131–3138. 10.1099/jgv.0.000626, PMID: 27902376

[ref14] Gunawan E. Utsumi T. Wahyuni R. M. Dinana Z. Sudarmo S. M. Shoji I.. (2019). Post-vaccinated asymptomatic rotavirus infections: a community profile study of children in Surabaya, Indonesia. J. Infect. Public Health 12, 625–629. 10.1016/j.jiph.2019.02.015, PMID: 30837151

[ref15] Hakim M. S. Nirwati H. Aman A. T. Soenarto Y. Pan Q. (2018). Significance of continuous rotavirus and norovirus surveillance in Indonesia. World J. Pediatr. 14, 4–12. 10.1007/s12519-018-0122-1, PMID: 29446040

[ref16] Jain S. Thakur N. Grover N. Vashistt J. Changotra H. (2016). Prevalence of rotavirus, norovirus and enterovirus in diarrheal diseases in Himachal Pradesh, India. Virus Disease. 27, 77–83. 10.1007/s13337-016-0303-2, PMID: 26925447 PMC4758311

[ref17] Khamrin P. Tran D. N. Chan-it W. Thongprachum A. Okitsu S. Maneekarn N.. (2011). Comparison of the rapid methods for screening of group a rotavirus in stool samples. J. Trop. Pediatr. 57, 375–377. 10.1093/tropej/fmq101, PMID: 21030457

[ref18] Komoto S. Ide T. Negoro M. Tanaka T. Asada K. Umemoto M.. (2018). Characterization of unusual DS-1-like G3P[8] rotavirus strains in children with diarrhea in Japan. J. Med. Virol. 90, 890–898. 10.1002/jmv.25016, PMID: 29315643

[ref19] Komoto S. Tacharoenmuang R. Guntapong R. Ide T. Tsuji T. Yoshikawa T.. (2016). Reassortment of human and animal rotavirus gene segments in emerging DS-1-like G1P[8] rotavirus strains. PLoS One 11:e0148416. 10.1371/journal.pone.0148416, PMID: 26845439 PMC4742054

[ref20] Lestari F. B. Vongpunsawad S. Wanlapakorn N. Poovorawan Y. (2020). Rotavirus infection in children in Southeast Asia 2008–2018: disease burden, genotype distribution, seasonality, and vaccination. J. Biomed. Sci. 27:66. 10.1186/s12929-020-00649-8 32438911 PMC7239768

[ref21] Luchs A. da Costa A. C. Cilli A. Komninakis S. Carmona R. Boen L.. (2019). Spread of the emerging equine-like G3P[8] DS-1-like genetic backbone rotavirus strain in Brazil and identification of potential genetic variants. J. Gen. Virol. 100, 7–25. 10.1099/jgv.0.001171, PMID: 30457517

[ref22] Matthijnssens J. Ciarlet M. McDonald S. M. Attoui H. Bányai K. Brister J. R.. (2011). Uniformity of rotavirus strain nomenclature proposed by the rotavirus classification working group (RCWG). Arch. Virol. 156, 1397–1413. 10.1007/s00705-011-1006-z, PMID: 21597953 PMC3398998

[ref23] Mukhopadhya I. Murdoch H. Berry S. Hunt A. Iturriza-Gomara M. Smith-Palmer A.. (2017). Changing molecular epidemiology of rotavirus infection after introduction of monovalent rotavirus vaccination in Scotland. Vaccine 35, 156–163. 10.1016/j.vaccine.2016.11.028, PMID: 27876201

[ref24] Nirwati H. Donato C. M. Ikram A. Aman A. T. Wibawa T. Kirkwood C. D.. (2019). Phylogenetic and immunoinformatic analysis of VP4, VP7, and NSP4 genes of rotavirus strains circulating in children with acute gastroenteritis in Indonesia. J. Med. Virol. 91, 1776–1787. 10.1002/jmv.25527, PMID: 31243786

[ref25] Nirwati H. Hakim M. S. Aminah S. Dwija I. Pan Q. Aman A. T. (2017). Identification of rotavirus strains causing diarrhoea in children under five years of age in Yogyakarta, Indonesia. Malays J. Med. Sci. 24, 68–77. 10.21315/mjms2017.24.2.9, PMID: 28894406 PMC5566064

[ref26] Parwata W. S. S. Sukardi W. Wahab A. Soenarto Y. (2016). Prevalence and clinical characteristics of rotavirus diarrhea in Mataram, Lombok, Indonesia. Paediatr. Indones. 56, 118–123. 10.14238/pi56.2.2016.118-23

[ref27] Putnam S. D. Sedyaningsih E. R. Listiyaningsih E. Pulungsih S. P. Komalarini S. Soenarto Y.. (2007). Group a rotavirus-associated diarrhea in children seeking treatment in Indonesia. J. Clin. Virol. 40, 289–294. 10.1016/j.jcv.2007.09.005, PMID: 17977785

[ref28] Radji M. Putman S. D. Malik A. Husrima R. Listyaningsih E. (2010). Molecular characterization of human group a rotavirus from stool samples in young children with diarrhea in Indonesia. Southeast Asian J. Trop. Med. Public Health 41, 341–346. PMID: 20578517

[ref29] Roczo-Farkas S. Kirkwood C. D. Bines J. E. and the Australian Rotavirus Surveillance Group (2016). Australian rotavirus surveillance program annual report, 2015. Commun. Dis. Intell. Q. Rep. 40, E527–E538. PMID: 28043228 10.33321/cdi.2016.40.60

[ref30] Rotavirus Classification Working Group (2019). List of accepted genotypes. Available at: https://rega.kuleuven.be/cev/viralmetagenomics/virus-classification/newgenotypes (Accessed April 2, 2019).

[ref31] Salim H. Karyana I. P. Sanjaya-Putra I. G. Budiarsa S. Soenarto Y. (2014). Risk factors of rotavirus diarrhea in hospitalized children in Sanglah hospital, Denpasar: a prospective cohort study. BMC Gastroenterol. 14:54. 10.1186/1471-230X-14-54 24669783 PMC3986934

[ref32] Santos N. Hoshino Y. (2005). Global distribution of rotavirus serotypes/genotypes and its implication for the development and implementation of an effective rotavirus vaccine. Rev. Med. Virol. 15, 29–56. 10.1002/rmv.448, PMID: 15484186

[ref33] Soenarto Y. Aman A. T. Bakri A. Waluya H. Firmansyah A. Kadim M.. (2009). Burden of severe rotavirus diarrhea in Indonesia. J. Infect. Dis. 200, S188–S194. 10.1086/605338, PMID: 19821711

[ref34] Sudarmo S. M. Shigemura K. Athiyyah A. F. Osawa K. Wardana O. P. Darma A.. (2015). Genotyping and clinical factors in pediatric diarrhea caused by rotaviruses: one-year surveillance in Surabaya, Indonesia. Gut. Pathog. 7:3. 10.1186/s13099-015-0048-2, PMID: 25793014 PMC4365806

[ref35] Tamura K. Dudley J. Nei M. Kumar S. (2007). MEGA4: molecular evolutionary genetics analysis (MEGA) software version 4.0. Mol. Biol. Evol. 24, 1596–1599. 10.1093/molbev/msm092, PMID: 17488738

[ref36] Tran A. Talmud D. Lejeune B. Jovenin N. Renois F. Payan C.. (2010). Prevalence of rotavirus, adenovirus, norovirus, and astrovirus infections and coinfections among hospitalized children in northern France. J. Clin. Microbiol. 48, 1943–1946. 10.1128/JCM.02181-09, PMID: 20305010 PMC2863921

[ref37] Troeger C. Khalil I. A. Rao P. C. Cao S. Blacker B. F. Ahmed T.. (2018). Rotavirus vaccination and the global burden of rotavirus diarrhea among children younger than 5 years. JAMA Pediatr. 172, 958–965. 10.1001/jamapediatrics.2018.1960, PMID: 30105384 PMC6233802

[ref38] Ugboko H. U. Nwinyi O. C. Oranusi S. U. Oyewale J. O. (2020). Childhood diarrhoeal diseases in developing countries. Heliyon. 6:e03690. 10.1016/j.heliyon.2020.e03690, PMID: 32322707 PMC7160433

[ref39] Utsumi T. Wahyuni R. M. Dinana Z. Gunawan E. Putra A. S. D. Mubawadi T.. (2020). G2P[4] rotavirus outbreak in Belu, East Nusa Tenggara Province, Indonesia, 2018. J. Infect. Public Health 13, 1592–1594. 10.1016/j.jiph.2020.05.002, PMID: 32475806

[ref40] Utsumi T. Wahyuni R. M. Doan Y. H. Dinana Z. Soegijanto S. Fujii Y.. (2018). Equine-like G3 rotavirus strains as predominant strains among children in Indonesia in 2015-2016. Infect. Genet. Evol. 61, 224–228. 10.1016/j.meegid.2018.03.027, PMID: 29614325

